# Ibrutinib versus temsirolimus: 3-year follow-up of patients with previously treated mantle cell lymphoma from the phase 3, international, randomized, open-label RAY study

**DOI:** 10.1038/s41375-018-0023-2

**Published:** 2018-02-02

**Authors:** S Rule, W Jurczak, M Jerkeman, C Rusconi, M Trneny, F Offner, D Caballero, C Joao, M Witzens-Harig, G Hess, I Bence-Bruckler, S-G Cho, C Thieblemont, W Zhou, T Henninger, J Goldberg, J Vermeulen, M Dreyling

**Affiliations:** 1 Plymouth University Medical School, Plymouth, UK; 2 Department of Hematology, Jagiellonian University, Krakow, Poland; 3 Skånes University Hospital, Lund University, Lund, Sweden; 4 Hematology Division, Hematology and Oncology Department, Niguarda Cancer Center, Niguarda Hospital, Milan, Italy; 5 Ist Dept Medicine, Charles University General Hospital, Prague, Czech Republic; 6 Departement Oncologie, UZ Gent, Ghent, Belgium; 7 Instituto Biosanitario de Salamanca, Hospital Clinico Universitario Salamanca, Salamanca, Spain; 8 Institutto Português de Oncologia de Lisboa, Portugal and Champalimaud Centre for the Unknown, Hematology, Lisbon, Portugal; 9 Klinikum der Ruprechts-Karls-Universität Heidelberg, Med. Klinik u. Poliklinik V, Heidelberg, Germany; 10 Department of Hematology, Oncology and Pneumology, University Medical School of the Johannes Gutenberg University, Mainz, Germany; 11 The Ottawa Hospital, General Campus, Ottawa, ON Canada; 12 Seoul St. Mary’s Hospital, Seocho-gu, Seoul, South Korea; 13 APHP, Saint-Louis Hospital, Hemato-oncology, Diderot University, Paris, France; 14 Janssen Research & Development, Raritan, NJ USA; 15 Janssen Research & Development, Leiden, The Netherlands; 16 Department of Medicine III, Klinikum der Universität München, LMU, Munich, Germany

Mantle cell lymphoma (MCL) is an aggressive B-cell malignancy with a reported median overall survival (OS) of 3–5 years [1]. Most patients relapse after first-line therapy and have a poor prognosis [1]. Regulatory approval of ibrutinib has provided a much needed therapeutic option for patients with relapsed or refractory (R/R) MCL [2], with ibrutinib becoming a preferred standard of care in current guidelines [3, 4]. The randomized, open-label phase 3 RAY study (NCT01646021) was key in confirming the efficacy and safety of ibrutinib, with ibrutinib (*N* = 139) showing significantly improved progression-free survival (PFS) versus temsirolimus (*N* = 141) (primary analysis [20-month follow-up]: 14.6 vs. 6.2 months, hazard ratio [HR] 0.43, 95% confidence interval [CI]: 0.32–0.58) [5]. Here, we report extended follow-up data from the final analysis of the RAY study.

At this final analysis, after an almost doubled median study follow-up of 38.7 months, 33 patients (24%) in the ibrutinib group and no patients in the temsirolimus group remained on initially randomized treatment. Crossover to ibrutinib from the temsirolimus group was permitted for patients who had confirmed disease progression. Fifty-five patients in the temsirolimus group (39%) received subsequent ibrutinib (42 were included in the formal study crossover; 13 received ibrutinib outside of the study). Disease progression or relapse was the most common reason for discontinuing treatment for both groups (ibrutinib, 78 patients [56%]; temsirolimus, 66 patients [47%]). Fewer patients in the ibrutinib group (12 [9%]) than in the temsirolimus group (39 [28%]) discontinued treatment due to adverse events (AEs); eight patients in each arm discontinued due to death. Other reasons for discontinuation included refusing further treatment. Median duration of exposure was longer for ibrutinib than temsirolimus (ibrutinib, 14.4 months; temsirolimus, 3.0 months), as in the primary analysis.

**Fig. 1:**
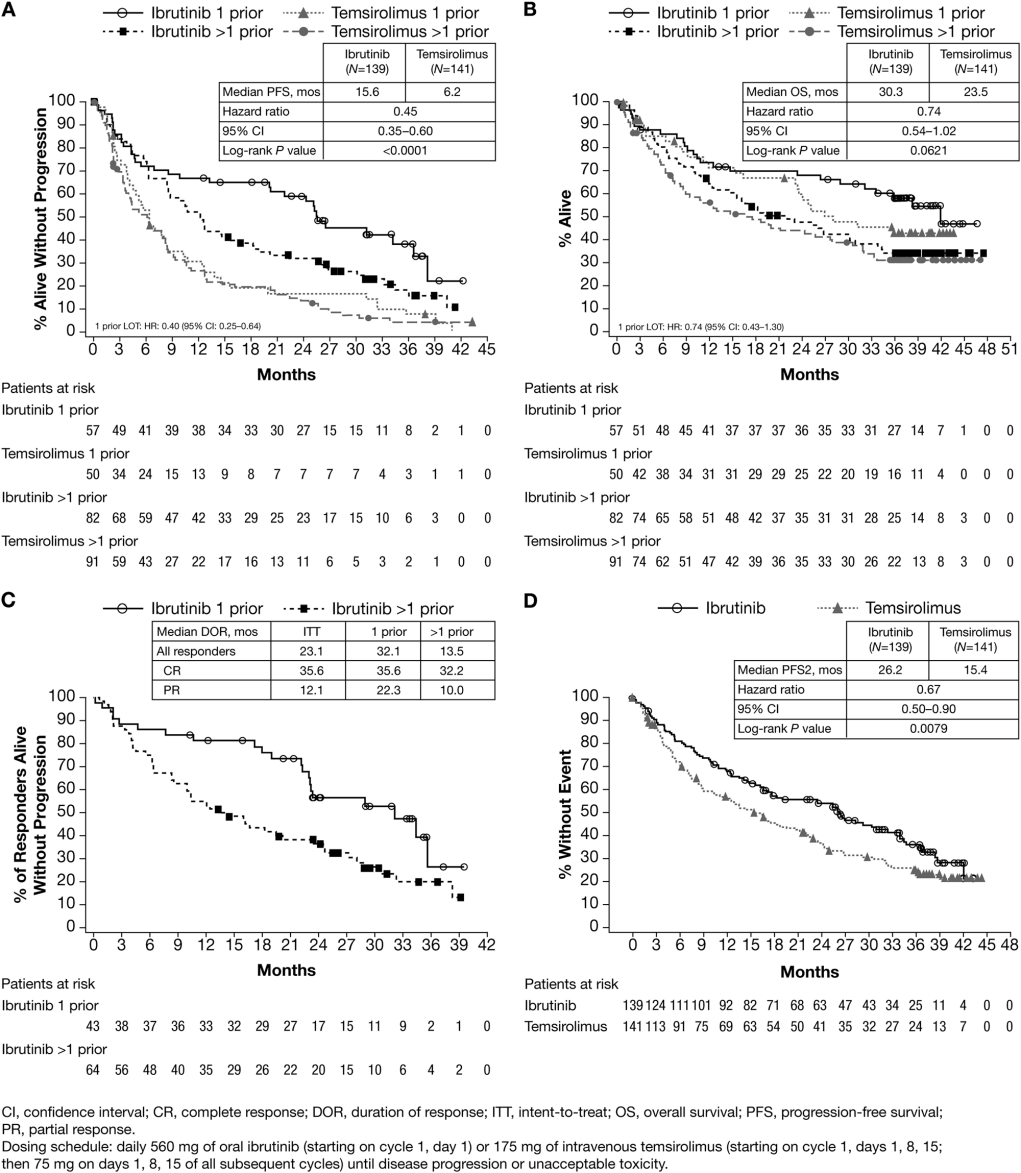
Efficacy end points in 3-year follow-up in RAY study: **a** Progression-free survival for ibrutinib and temsirolimus by prior line of therapy; **b** Overall survival for ibrutinib and temsirolimus by prior line of therapy; **c** Duration of clinical response by prior line of therapy in patients randomized to ibrutinib; **d** Time to second progression or death for ibrutinib and temsirolimus

Efficacy assessments at primary analysis by the Independent Review Committee showed high concordance with investigator assessment; at final analysis, all efficacy analyses were based on investigator assessment. With additional follow-up, median PFS remained significantly longer for ibrutinib than temsirolimus (15.6 vs. 6.2 months; HR 0.45 [95% CI 0.35–0.60]; *P* < 0.0001); consistent with the results of the primary analysis [5]. An exploratory post hoc analysis evaluated PFS by number of prior lines of therapy received (ibrutinib, 57 [41%] 1 prior line and 82 [59%] >1 prior line; temsirolimus, 50 [35%] 1 prior line and 91 [65%] >1 prior line). Median PFS for ibrutinib was significantly longer than temsirolimus regardless of the number of prior lines of treatment, and the difference in median PFS between ibrutinib-treated and temsirolimus-treated patients was greatest in those who received 1 prior line of therapy versus >1 (1 prior line, 25.4 vs. 6.2 months, respectively, HR 0.40 [95% CI 0.25–0.64] >1 prior line, 12.1 vs. 6.0 months respectively, HR 0.53 [95% CI 0.38–0.73]; Fig. 1a).

At the time of final analysis, 77 patients (55%) in the ibrutinib group and 83 (59%) in the temsirolimus group had died, with a trend toward improved OS in the patients randomized to receive ibrutinib versus temsirolimus (30.3 vs. 23.5 months, respectively; HR 0.74 [95% CI 0.54–1.02]; *P* = 0.0621). Median OS was longer for ibrutinib than temsirolimus regardless of the extent of prior treatment. However, similar to PFS, a more pronounced OS difference was observed between ibrutinib and temsirolimus treatment in those patients who had received 1 prior line of therapy (1 prior line, 42.1 vs. 27.0 months, respectively, HR 0.74 [95% CI 0.43–1.30]; >1 prior line, 22.1 vs. 17.0 months respectively, HR 0.86 [95% CI 0.59–1.25]; Fig. 1b).

Overall response rate (ORR) in the final analysis was consistent with the primary analysis (77% for ibrutinib vs. 47% for temsirolimus; odds ratio 4.27 [95% CI 2.47–7.39]; *P* < 0.0001), with a higher proportion of patients achieving a complete response (CR) with ibrutinib (23%) than with temsirolimus (3%). ORR results for ibrutinib were similar regardless of extent of prior treatment (75 vs. 78% for 1 prior line and >1 prior line, respectively). However, the CR rate was two-fold higher in patients treated with ibrutinib who received 1 prior line of therapy than those who received >1 prior line: 33 and 16%, respectively. Overall median duration of response (DOR) was 23.1 months (95% CI 16.2–28.1) with ibrutinib and 6.3 months (95% CI 4.7–8.6) with temsirolimus. Patients who achieved a CR on ibrutinib had a longer median DOR than patients who achieved a partial response (PR) (35.6 [*N* = 32] vs. 12.1 months [*n* = 75]; Fig. 1c). While DOR for patients achieving CR with ibrutinib remained consistent regardless of the extent of prior treatment (35.6 [*N* = 19] vs 32.2 months [*N* = 13] for 1 and >1 prior line of therapy, respectively), the DOR for patients achieving PR decreased with increasing lines of prior therapy (22.3 [*N* = 24] vs. 10.0 months [*N* = 51], respectively, for those who had received 1 vs. >1 prior line of therapy). Therefore, DOR for complete responders with only 1 prior line was more than three times longer than for partial responders with >1 prior line of therapy.

Consistent with the primary analysis, the most common treatment-emergent AEs (TEAEs) of any grade were diarrhea (33%), fatigue (24%), and cough (23%) in the ibrutinib group, and thrombocytopenia (56%), anemia (44%), and diarrhea (31%) in the temsirolimus group. Despite longer treatment exposure in the ibrutinib group versus the temsirolimus group, the frequency of grade ≥3 TEAEs (75 vs. 87%), serious AEs of any grade (57 vs. 60%) and AEs leading to discontinuation (17 vs. 32%) were lower in the ibrutinib group than in the temsirolimus group, respectively. The most common grade ≥3 TEAEs for both groups were hematological in nature and were less frequently reported in the ibrutinib group than the temsirolimus group, respectively: neutropenia (13 vs. 17%), thrombocytopenia (9 vs. 43%) and anemia (9 vs. 20%) (Table 1). The rate of any grade bleeding was 40 and 33% in the ibrutinib and temsirolimus groups, respectively. The rate of grade ≥3 bleeding was 9% in the ibrutinib group and 5% in the temsirolimus group, with exposure-adjusted rates being lower in the ibrutinib group (0.455 events per 100 patient-months) versus the temsirolimus group (0.785 events per 100 patient-months). A higher rate of grade ≥3 atrial fibrillation was observed in the ibrutinib group (5%) versus the temsirolimus group (1%); exposure-adjusted rates were similar for both groups (0.272 events per 100 patient-months for ibrutinib; 0.221 events per 100 patient-months for temsirolimus).

**Table 1:** Treatment-emergent adverse events (AEs) in ≥20% of patients in either treatment arm

Safety population	Ibrutinib (*N* = 139)		Temsirolimus (*N* = 139)	
AE (%)	Any grade	Grade ≥3	Any grade	Grade ≥3
Hematological				
Thrombocytopenia	18.0	9.4	56.1	43.2
Anemia	19.4	8.6	43.9	20.1
Neutropenia	15.8	12.9	26.6	17.3
Non-hematological				
Diarrhea	33.1	3.6	30.9	4.3
Fatigue	23.7	5.0	28.8	7.2
Cough	23.0	0.7	22.3	0.0
Upper respiratory tract infection	20.1	2.2	11.5	0.7
Pyrexia	18.7	0.7	20.9	2.2
Nausea	14.4	0.0	21.6	0.0
Peripheral edema	13.7	0.0	23.7	2.2
Epistaxis	9.4	0.7	23.7	1.4
Stomatitis	2.9	0.0	20.9	3.6

With longer-term follow-up, the data support a sustained clinical benefit of ibrutinib. Median time to next treatment (TTNT) was longer for patients in the ibrutinib group versus the temsirolimus group (31. 8 vs. 11.6 months; HR 0.33 [95% CI 0.24–0.46]; *P* < 0.0001). Moreover, median time from randomization to progression or death after subsequent therapy (PFS2) was longer for ibrutinib than temsirolimus (26.2 vs. 15.4 months; HR 0.67 [95% CI 0.50–0.90]; *P* = 0.0079; Fig. 1d).

Nearly half (*N* = 29; 46%) of 63 patients randomized to ibrutinib who received subsequent anticancer therapy on study were treated with rituximab-based chemotherapy. In these 29 patients, following treatment with ibrutinib, the ORR with rituximab-based chemotherapy was 41% (24% CR [*N* = 7]; 17% PR [*N* = 5]); response was missing or not evaluable in 11 patients. Fifteen of these 29 patients were treated specifically with bendamustine-rituximab following ibrutinib (ORR 53%; 40% CR [*N* = 6], 13% PR [*N* = 2]); response was missing or not evaluable in six patients.

In conclusion, longer-term follow-up from the final analysis of the RAY study supports the initial report, demonstrating significant improvement in ORR and PFS with ibrutinib over temsirolimus in patients with R/R MCL. At the final analysis, OS showed a trend in favor of ibrutinib versus temsirolimus (30.3 vs. 23.5 months; HR 0.74 [95% CI 0.54–1.02], *P* = 0.0621). In the initial analysis, number of previous lines of therapy was identified as a prognostic factor [5]. With longer follow-up this was evident, with patients who had received 1 prior line of therapy benefiting the most from the use of ibrutinib. More patients were able to achieve a CR (33 vs. 16%), and those achieving a PR had a longer DOR (22.3 vs. 10.0 months) when using ibrutinib after 1 versus >1 prior line of therapy. In ibrutinib patients with 1 prior line of therapy, this resulted in a doubling of PFS versus ibrutinib patients with >1 prior line of therapy (25.4 vs. 12.1 months) and an almost 15-month improvement of OS versus temsirolimus patients with 1 prior line of therapy (42.1 vs. 27.0 months). These data from the RAY study, irrespective of the number of prior lines of therapy, compare favorably to the results from pivotal clinical trials of other single agents in R/R MCL (e.g., bortezomib, lenalidomide, and temsirolimus), the use of which was associated with median PFS of 4–5 months, median OS of 13–19 months, and ORRs of 22–33% [6–9]. Given that these findings support earlier use of ibrutinib in the relapsed/refractory setting, a relevant clinical question is whether patients can be successfully treated after progression on ibrutinib. Here, we show that patients could be successfully rescued post ibrutinib therapy with rituximab-based chemotherapy (ORR = 41%), including bendamustine-rituximab (ORR = 53%). Importantly, longer follow-up revealed no new late or cumulative toxicities, supporting the overall well-tolerated safety profile for ibrutinib [5]. The significant improvements in PFS2 provide further evidence that ibrutinib benefit is maintained beyond subsequent lines of treatment. Collectively, these results support the role of ibrutinib in the treatment of previously treated MCL. Emerging data suggest that ibrutinib may also have a role in treatment-naïve MCL [10], with multiple phase 3 studies underway (e.g., ENRICH [EudraCT 2015-000832-13], SHINE [NCT01776840], and TRIANGLE [NCT02858258]).
